# An unusual variation of deltoid muscle

**DOI:** 10.4103/0973-6042.42201

**Published:** 2008

**Authors:** Haldun O. Kamburoğlu, Omer F. Boran, Mustafa F. Sargon, Abdullah Keçik

**Affiliations:** Department of Plastic Reconstructive and Hand Surgery, Hacettepe University, Faculty of Medicine, Ankara, Turkey; 1 Department of Anatomy, Hacettepe University Faculty of Medicine, Ankara, Turkey

**Keywords:** Deltoid flap, deltoid muscle, variation

## Abstract

An unusual anatomic variation of the deltoid muscle was found in a 45-year-old female cadaver during dissection of the right upper extremity. The posterior fibers of the right deltoid muscle were enclosed in a distinct fascial sheet and the deltoid muscle was seen to arise from the middle 1/3 of the medial border of the scapula. There was no accompanying vascular or neural anomaly of the deltoid muscle. To the best of our knowledge, unilateral posterior separation of the deltoid muscle with a distinct fascia has not been described previously. While dissecting deltoid, posterior deltoid, or scapular flaps, the surgeon needs to look out for this variation because it may cause confusion.

## INTRODUCTION

The deltoid muscle derives from the dorsal muscle mass of the limb bud which is formed by somatic mesoderm during the fifth intrauterine week.[1] Variations and anomalies of the deltoid muscle are not common. We report a very rare variation of the deltoid muscle because of its clinical and anatomical relevance. Bilateral separation of the posterior fibers with a fascia has been described in 1993.[2] However, to the best of our knowledge, unilateral separation of the posterior fibers of the deltoid muscle with a distinct fascia has not been described previously.

## CASE REPORT

**Figure 1:**
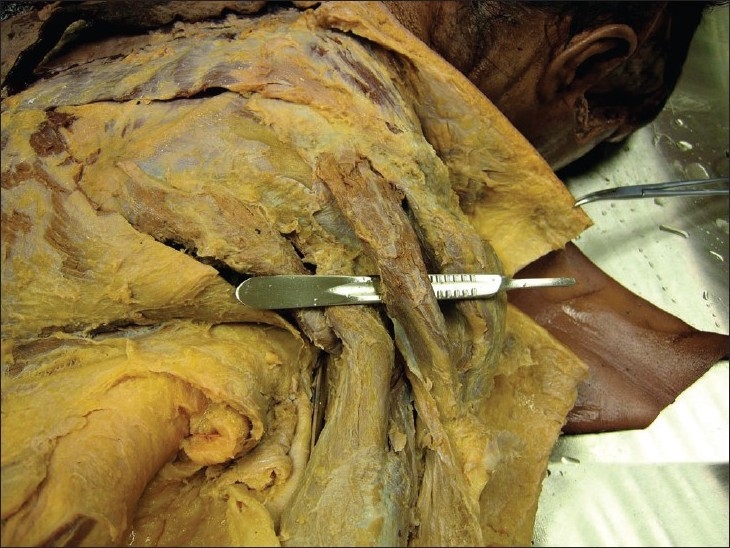
The cadaveric photograph of the accessory deltoid muscle

During the gross anatomic dissection of the right upper extremity of a 45-year-old female cadaver, we observed an unusual anatomic variation of the deltoid muscle. The posterior fibers of the right deltoid muscle were enclosed in a distinct fascial sheath [Figure 1]. In addition, the deltoid was seen to arise from the middle 1/3 of the medial border of the scapula. No variation in the left deltoid muscle was found. We made further dissections to check the vascular and neural structures. Although the posterior fibers were enclosed in a separate fascial sheath and arose from the middle 1/3 of the medial border of the scapula, its innervation was by the posterior branch of the axillary nerve and its blood supply was through the posterior circumflex humeral artery. There was no accompanying vascular or neural anomaly of the deltoid muscle. There was no other muscular anomaly in the muscular system of the body.

## DISCUSSION

The deltoid muscle arises from the anterior border and upper surface of the lateral third of the clavicle, the lateral margin and upper surface of the acromion, and the lower edge of the posterior border of the spine of the scapula. The insertion is into the deltoid tubercle on the middle of the lateral side of the body of the humerus.[3] It is innervated by the fifth and sixth cervical spinal nerves through the axillary nerve. Its vascular supply is through the acromial and deltoid branches of the thoracoacromial artery, the anterior and posterior circumflex humeral arteries, and the deltoid branch of the profunda brachii.[3]

The continuation of the fibers of the deltoid muscle into the trapezius; fusion with the pectoralis major; and the presence of additional slips from the vertebral border of the scapula, infraspinous fascia, and the axillary border of scapula are the commonly reported variations of the deltoid muscle.[3]

The myogenic cells coalesce into two muscle masses during the fifth intrauterine week.[1] One is the precursor of the flexor muscles the other is the precursor of the extensor muscles. These common muscle masses then split into anatomically recognizable precursors of the definitive muscles of the limb. There is little data about the mechanism of this splitting.[4] The deltoid muscle derives from the dorsal muscle mass.[1] An accessory deltoid may be developed because of incorrect splitting of the dorsal muscle mass.

Clinically fasciocutaneus, musculocutaneus or muscular deltoid and posterior deltoid flaps are especially used in; tetraplegia (by a transfer to triceps), radionecrotic defects situated over the glenohumeral joint, reconstruction of extremity, rotator cuff tears, and oral cavity. While elevating musculocutaneus or muscular deltoid and posterior deltoid flaps, the surgeon must be alert to the possibility of this variation's presence because it may cause confusion when dissecting the borders. Similarly, while elevating fasciocutaneus deltoid and posterior deltoid flaps or a scapular flap (either transverse or parascapular), an accessory deltoid may be confused with the teres major muscle because of its location and its distinct fascia and as a result of this the dissection of the pedicle can be much more difficult. To conclude, the variations possible in this region should be kept in mind during any surgery.
